# Nominal Equational Problems

**DOI:** 10.1007/978-3-030-71995-1_2

**Published:** 2021-03-23

**Authors:** Mauricio Ayala-Rincón, Maribel Fernández, Daniele Nantes-Sobrinho, Deivid Vale

**Affiliations:** 8 University of Oxford, Oxford, UK; 9 Sorbonne Université - LIP6, Paris, France; 10 Departments of Computer Science and Mathematics, Universidade de Brasília, Brasília D.F., Brazil; 11 Department of Informatics, King’s College London, London, UK; 12 Department of Software Science, Radboud University Nijmegen, Nijmegen, The Netherlands

**Keywords:** Nominal syntax, Unification, Disunification

## Abstract

We define *nominal equational problems* of the form $\exists \overline{W} \forall \overline{Y} : P$, where $P$ consists of conjunctions and disjunctions of equations $s\approx _\alpha t$, freshness constraints $a\#t$ and their negations: $s \not \approx _\alpha t$ and , where $a$ is an atom and $s, t$ nominal terms. We give a general definition of solution and a set of simplification rules to compute solutions in the nominal ground term algebra. For the latter, we define notions of solved form from which solutions can be easily extracted and show that the simplification rules are sound, preserving, and complete. With a particular strategy for rule application, the simplification process terminates and thus specifies an algorithm to solve nominal equational problems. These results generalise previous results obtained by Comon and Lescanne for first-order languages to languages with binding operators. In particular, we show that the problem of deciding the validity of a first-order equational formula in a language with binding operators (i.e., validity modulo $\alpha$-equality) is decidable.

### 1 Introduction

*Nominal unification* [23] is the problem of solving equations modulo $\alpha$-equivalence. A solution consists of a substitution and a freshness context $\nabla$, i.e., a set of primitive constraints of the form $a\#X$ (read: "$a$ is fresh for $X$"), which intuitively means that $a$ cannot occur free in the instances of $X$. Nominal unification is decidable and unitary [23], and efficient algorithms exist [5,17], which can be used to solve problems of the form $\exists \overline{X} \, (\bigwedge \Delta_i \vdash s_i \approx_\alpha t_i)$, where $s_i$, $t_i$ are nominal terms with variables $\overline{X}$ and $\Delta_i$ is a freshness context.

⋆ First author partially founded by PrInt MAT-UnB-CAPES and CNPq grant numbers Ed 41/2017 and 07672/2017-4. Third author partially supported by DPI/UnB - 03/2020. Fourth author supported by NWO TOP project "Implicit Complexity through Higher Order Rewriting" (ICHOR), NWO 612.001.803/7571.

Similarly, nominal disunification is the problem of solving disequations i.e., negated equations of the form $s \not\approx_\alpha t$. An algorithm to solve *nominal constraint problems* of the form

$$\mathcal{P} := \exists \overline{X} \left( \left( \bigwedge \Delta_i \vdash s_i \approx_\alpha t_i \right) \wedge \left( \bigwedge \nabla_j \vdash p_j \not\approx_\alpha q_j \right) \right)$$

is available [1], which finds solutions in the nominal term algebra $\mathcal{T}(\Sigma, \mathbb{A}, \mathbb{X})$ by constructing suitable representation of the witnesses for the variables in $\mathcal{P}$.

Comon and Lescanne [10] investigated a more general version of this problem, called *equational problem*, in their words: "an equational problem is any first-order formula whose only predicate symbol is =", that is, it has the form $\exists w_1, \ldots, w_n \forall y_1, \ldots, y_m : P$ where $P$ is a *system*, i.e., an equation $s = t$, or a disequation $s \neq t$, or a disjunction of systems $\bigvee P_i$, or a conjunction of systems $\bigwedge P_i$, or a failure $\perp$, or success $\top$. The study of such problems was motivated by applications in pattern-matching for functional languages, sufficient completeness for term rewriting systems, negation in logic programming languages, etc.

In order to extend these applications to languages that offer support for binders and $\alpha$-equivalence following the nominal approach, such as $\alpha$Prolog [6], $\alpha$Kanren [4], $\alpha$LeanTAP [20], to nominal rewriting [14] and nominal (universal) algebra [15], in this paper we consider *nominal equational problems.*

Based on Comon and Lescanne's work, the nominal extension of a first-order equational problem is a formula $\mathcal{P} ::= \exists W_1 \ldots W_n \forall Y_1 \ldots Y_m : P$ where $P$ is a *nominal system*, i.e., a formula consisting of conjunctions and disjunctions of freshness, equality constraints, and their negations.

*Contributions.* This paper introduces nominal equational problems (`NEP`s) and presents simplification rules to find solutions in the ground nominal algebra. The simplification rules are shown to be terminating (by using a measure that strictly decreases with each rule application), and also sound and solution-preserving. The simplification process for `NEP`s is more challenging than in the syntactic case because it deals with two predicates ($\approx_\alpha$ and $\#$) and needs to consider the interaction between freshness and $\alpha$-equality constraints, and quantifiers. The elimination of universal quantifiers requires careful analysis since universal variables may occur in freshness constraints and in their negations. To make the process more manageable, we define a set of rules together with a strategy of application (specified by rule conditions) that simplifies the termination proof.

Finally, we show that the irreducible forms are either $\perp$ or problems from which a solution can be easily extracted. In particular, if the `NEP` consists only of existentially quantified conjunctions of freshness and $\alpha$-equality constraints, we obtain solved forms consisting of a substitution and a freshness context, as in the standard nominal unification algorithm [23].

*Related Work.* Comon and Lescanne [10] introduced first-order equational problems and studied their solutions in the algebra of rational trees, the initial term algebra, and the ground term algebra. A restricted version of equational problems, called disunification problems, which do not contain quantified variables, has been extensively studied in the first-order framework [8,3,11,2,22]. More recently, a nominal approach to disunification problems was proposed by Ayala et.al [1], including only conjunctions of equations and disequations and freshness constraints, without quantified variables. Here we generalise this previous work to deal with general formulas including disjunction, conjunction and negation of equations and freshness constraints, as well as existential and universal quantification over variables. To deal with negation of freshness, disjunctive formulas, and quantification we extend the semantic interpretation and design a different set of simplification rules as well as a more elaborated strategy for rule application.

Extensions of first-order equational problems modulo equational theories have also been considered. Although the problem of solving disequations modulo an equational theory is not even semi-decidable in general (as shown by Comon [7]), there are useful decidable and semi-decidable cases. For example, solvability of complement problems (a sub-class of equational problems) is decidable modulo theories with permutative operators (which include commutative theories) [9,13], and for linear complement problems solvability modulo associativity and commutativity is also decidable [16,19,12]. Buntine and Bürckert [3] solve systems of equations and disequations in equational theories with a finitary unification type. Fernández [11] shows that $E$-disunification is semi-decidable when the theory $E$ is presented by a ground convergent rewrite system, and gives a sound and complete $E$-disunification procedure based on narrowing. Baader and Schulz [2] show that solvability of disunification problems in the free algebra of the combined theory $E_1 \cup \ldots \cup E_n$ is decidable if solvability of disunification problems with linear constant restrictions in the free algebras of the theories $E_i (1 \leq i \leq n)$ is decidable. Lugiez [18] introduces higher-order disunification problems and gives some decidable cases for which equational problems can be extended to higher-order systems.

*Organisation.* Section 2 recalls the main concepts of nominal syntax and semantics. Section 3 introduces nominal equational problems and a notion of solution for such problems. Section 4 presents a rule-based procedure for solving `NEP`s, as well as soundness, preservation of solutions, and termination results. Section 5 shows that the simplification rules reach solved forms from which solutions can be easily extracted. Section 6 concludes and discusses future work.

### 2 Background

We assume the reader is familiar with nominal techniques and recall some concepts and notations that shall be used in the paper; for more details, see [14,21,23].

*Nominal Terms.* We fix countable infinite pairwise disjoint sets of *atoms* $\mathbb{A} = \{a, b, c, \ldots\}$ and *variables* $\mathbb{X} = \{X, Y, Z, \ldots\}$. Atoms follow the *permutative convention*: names $a, b$ range permutatively over $\mathbb{A}$. Therefore, they represent different objects. Let $\Sigma$ be a finite set of term-formers disjoint from $\mathbb{A}$ and $\mathbb{X}$ such that for each $f \in \Sigma$, a unique non-negative integer $n$ (the arity of $f$, written as $f : n$) is assigned. We assume there is at least one $f : n$ such that $n > 0$.

A *permutation* $\pi$ is a bijection $\mathbb{A} \to \mathbb{A}$ with finite domain, i.e., the set $\mathtt{dom}(\pi) := \{a \in \mathbb{A} \mid \pi(a) \neq a\}$ is finite. We shall represent permutations as lists of *swappings* $\pi = (a_1\ b_1)(a_2\ b_2)\dots(a_n\ b_n)$. The identity permutation is denoted by $\mathtt{id}$ and $\pi \circ \pi'$ the composition of $\pi$ and $\pi'$. The set $\mathbb{P}$ of all such permutations together with the composition operation form a group $(\mathbb{P}, \circ)$ and it will be denoted simply by $\mathbb{P}$. The *difference set* of $\pi$ and $\gamma$ is defined by $\mathtt{ds}(\pi, \gamma) = \{a \in \mathbb{A} \mid \pi(a) \neq \gamma(a)\}$.

**Definition 1 (Nominal Terms).** *The set $T(\Sigma, \mathbb{A}, \mathbb{X})$ of Nominal Terms, or just terms for short, is inductively defined by the following grammar:*

$$s, t, u ::= a \mid \pi \cdot X \mid [a]t \mid f(t_1, \dots, t_n),$$

*where $a$ is an* atom*, $\pi \cdot X$ is a moderated variable, $[a]t$ is the* abstraction *of $a$ in the term $t$, and $f(t_1, \dots, t_n)$ is a* function application *with $f \in \Sigma$ and $f : n$. A term is ground if it does not contain variables.*

In an abstraction $[a]t$, $t$ is the scope of the binder $[\cdot]$ and it *binds* all free occurrences of $a$ in $t$. An occurrence of an atom in a term is *free* if it is not under the scope of a binder. Notice that syntactical equality is not modulo $\alpha$-equivalence; for example, $[a]a \not\equiv [b]b$. We may denote $s \equiv t$ by $s = t$ with the same intended meaning and $\tilde{t}$ abbreviates an ordered sequence $t_1, \dots, t_n$ of terms.

*Example 1.* Let $\Sigma_\lambda := \{\mathtt{lam} : 1, \mathtt{app} : 2\}$ be a signature for the $\lambda$-calculus. Using atoms to represent variables, $\lambda$-expressions are generated by the grammar:

$$e ::= a \mid \mathtt{lam}([a]e) \mid \mathtt{app}(e, e)$$

As usual, we sugar $\mathtt{app}(s, t)$ to $s\,t$ and $\mathtt{lam}([a]s)$ to $\lambda[a]s$. The following are examples of nominal terms: $(\lambda[a]a)\,X$ and $(\lambda[a](\lambda[b]b\,a)\ c)\,d$.

We inductively extend the action of a permutation $\pi$ to a term $t$, denoted as $\pi \cdot t$, by setting: $\pi \cdot a = \pi(a), \pi \cdot (\pi' \cdot X) = (\pi \circ \pi') \cdot X, \pi \cdot ([a]t) = [\pi(a)](\pi \cdot t)$, and $\pi \cdot f(\tilde{t}) = f(\pi \cdot \tilde{t})$.

*Substitutions*, ranging over $\sigma, \gamma, \tau \dots$, are maps (with finite domain) from variables to terms. The *action of a substitution* $\sigma$ on a term $t$, denoted $t\sigma$, is inductively defined by: $a\sigma = a, (\pi \cdot X)\sigma = \pi \cdot (X\sigma)$, $([a]t)\sigma = [a](t\sigma)$ and $f(t_1, \dots, t_n)\sigma = f(t_1\sigma, \dots, t_n\sigma)$. Notice that $t(\sigma\gamma) = (t\sigma)\gamma$.

**Definition 2 (Positions and subterms).** *Let $s$ be a nominal term. The set $\mathtt{Pos}(s)$ of* positions *in $s$ is a set of strings over positive integers defined inductively below. Additionally, $s|_p$ denotes the subterm of $s$ at position $p$ and $s(p)$ denotes the symbol at position $p$.*

- *If $s = a$ or $s = \pi \cdot X$, then $\mathtt{Pos}(s) = \{\epsilon\}$ and $s|_\epsilon = s$;*
- *if $s = [a]t$ then $\mathtt{Pos}(s) = \{\epsilon\} \cup \{1 \cdot p \mid p \in \mathtt{Pos}(t)\}$, $s|_\epsilon = s$ and $s|_{1\cdot p} = t|_p$;*
- *if $s = f(s_1, \dots, s_n)$ then $\mathtt{Pos}(s) = \{\epsilon\} \cup \bigcup_{i=1}^n \{i \cdot p \mid p \in \mathtt{Pos}(s_i)\}$, $s|_\epsilon = s$ and $s|_{i\cdot p} = s_i|_p$.*

*Freshness and $\alpha$-equality.* A *nominal equation* is the symbol $\top$ or an expression $s \approx_\alpha t$ where $s$ and $t$ are nominal terms. A *trivial equation* is either $s \approx_\alpha s$ or $\top$. *Freshness constraints* have the form $a\#t$ where $a$ is an atom and $t$ a term. A *freshness context* is a finite set of *primitive* freshness constraints of the form $a\#X$, we use $\Delta, \nabla$, and $\Gamma$ to denote them. We extend the notation to sets of atoms: $A\#X$ denotes that $a\#X$ for every $a \in A$.

$\alpha$-derivability is given by the deduction rules in Figure 1, which define an *equational theory* called CORE.

$$\frac{}{\nabla \vdash a\#b}\ (\#\text{-ax}) \qquad \frac{\pi^{-1}(a)\#X \in \nabla}{\nabla \vdash a\#\pi \cdot X}\ (\#\text{-var}) \qquad \frac{}{\nabla \vdash a\#[a]t}\ (\#\text{-abs-a})$$

$$\frac{\nabla \vdash a\#t}{\nabla \vdash a\#[b]t}\ (\#\text{-abs-b}) \qquad \frac{\nabla \vdash a\#t_1 \quad \cdots \quad \nabla \vdash a\#t_n}{\nabla \vdash a\#f(t_1, \ldots t_n)}\ (\#\text{-f})$$

$$\frac{}{\nabla \vdash a \approx_\alpha a}\ (\text{ax}) \qquad \frac{\mathtt{ds}(\pi, \pi')\#X \in \nabla}{\nabla \vdash \pi \cdot X \approx_\alpha \pi' \cdot X}\ (\text{var}) \qquad \frac{\nabla \vdash t \approx_\alpha t'}{\nabla \vdash [a]t \approx_\alpha [a]t'}\ (\text{abs-a})$$

$$\frac{\nabla \vdash t \approx_\alpha (a\, a') \cdot t' \qquad \nabla \vdash a\#t'}{\nabla \vdash [a]t \approx_\alpha [a']t'}\ (\text{abs-b}) \quad \frac{\nabla \vdash t_1 \approx_\alpha t'_1 \quad \cdots \quad \nabla \vdash t_n \approx_\alpha t'_n}{\nabla \vdash f(t_1, \ldots t_n) \approx_\alpha f(t'_1, \ldots, t'_n)}\ (\text{f})$$

**Fig. 1.** CORE freshness and $\alpha$-equality rules.

- Write $\nabla \vdash a\#t$ when there exists a derivation of $\nabla \vdash a\#t$.
  The judgement $\nabla \vdash a\#t$ intuitively means that using freshness constraints from $\nabla$ as assumptions $a$ does not occur free in $t$.
- Write $\nabla \vdash s \approx_\alpha t$ when there exists a derivation of $\nabla \vdash s \approx_\alpha t$.
  The judgement $\nabla \vdash s \approx_\alpha t$ intuitively means that using freshness constraints from $\nabla$ as assumptions $s$ is $\alpha$-equivalent to $t$.

*Semantic Notions.* Nominal equational theory has a natural semantic denotation in *nominal sets* since we can easily interpret freshness and abstraction.

A $\mathbb{P}$-set $X$ is an ordinary set equipped with an action in $\mathbb{P} \times X \to X$ (written as $\pi \cdot x$) such that $\mathtt{id} \cdot x = x$ and $\pi \cdot (\pi' \cdot x) = (\pi \circ \pi') \cdot x$. A set of atoms $A \subset \mathbb{A}$ *supports* $x \in X$ iff for all permutations $\pi \in \mathbb{P}$ fixing every element of $A$ $\cdot$ acts trivially on $x$ via $\pi$, i.e., if $\pi(a) = a$ for all $a \in A$ then $\pi \cdot x = x$. *Semantic freshness* is defined in terms of support as follows: an atom $a$ is fresh for $x \in X$ iff $a \notin \mathtt{supp}(x)$. We denote this by writing $a\#_{\mathsf{sem}}x$. A *nominal set* is a $\mathbb{P}$-set such that every element is finitely supported.

To build an algebraic ground term-model of CORE, we fix the set $G$ consisting of equivalence classes of provable $\alpha$-equivalent ground terms. More precisely, given a ground term $g$, the class $\overline{g}$ is the set of ground terms $g'$ for which there exist a derivation $\vdash g \approx_\alpha g'$. Note that $G$ is a nominal set by defining the natural action: $\pi \cdot \overline{g} = \overline{\pi \cdot g}$. Each function symbol $f \in \Sigma$ is interpreted by an *equivariant function* $f^{\mathcal{I}}$ mapping $(\overline{t_1}, \ldots, \overline{t_n}) \mapsto \overline{f(t_1, \ldots, t_n)}$ and abstractions $[a]t$ are interpreted by an equivariant function $[\_]\_$ in $\mathbb{A} \times G \to G$ such that $a\#_{\mathsf{sem}}[a]g$ always.

Signature interpretation is homomorphically extended to the set of terms as follows: Fix a *valuation function* $\varsigma$ that assigns to every variable $X \in \mathbb{X}$ an element of $G$. The interpretation of a term $t$ under $\varsigma$, $[\![t]\!]_\varsigma$, is defined as:

$$[\![a]\!]_\varsigma = \overline{a} \qquad [\![\pi \cdot X]\!]_\varsigma = \pi \cdot \varsigma(X) \qquad [\![[a]t]\!]_\varsigma = [\overline{a}][\![t]\!]_\varsigma$$
$$[\![f(t_1, \ldots, t_n)]\!]_\varsigma = f^{\mathcal{I}}([\![t_1]\!]_\varsigma, \ldots, [\![t_n]\!]_\varsigma)$$

**Definition 3 (Validity under $\varsigma$).** *Let $\mathcal{A}$ be any infinite subalgebra of* CORE *with domain $A$ and $\varsigma$ a valuation function assigning for every variable $X \in \mathbb{X}$ an element of $A$. We say that:*

1. *$[\![a\#t]\!]_\varsigma$ (resp. $[\![t \approx_\alpha u]\!]_\varsigma$) is valid if $a\#_{\mathsf{sem}} [\![t]\!]_\varsigma$ (resp. $[\![t]\!]_\varsigma = [\![u]\!]_\varsigma$).*
2. *$[\![\nabla]\!]_\varsigma$ is valid when $a\#_{\mathsf{sem}}\varsigma(X)$ for each $a\#X \in \nabla$.*
3. *$[\![\nabla \vdash a\#t]\!]_\varsigma$ is valid when the validity of $[\![\nabla]\!]_\varsigma$ implies $a\#_{\mathsf{sem}} [\![t]\!]_\varsigma$, and*
4. *$[\![\nabla \vdash t \approx_\alpha u]\!]_\varsigma$ is valid when the validity of $[\![\nabla]\!]_\varsigma$ implies $[\![t]\!]_\varsigma = [\![u]\!]_\varsigma$.*

*Write $\nabla \models s \approx_\alpha t$ (resp. $\nabla \models a\#t$) when $[\![\nabla \vdash s \approx_\alpha t]\!]_\varsigma$ (resp. $[\![\nabla \vdash a\#t]\!]_\varsigma$) is valid for any valuation $\varsigma$.*

A model of a nominal theory is an interpretation that validates all of its axiomatic judgements $\nabla \vdash s \approx_\alpha t$. It is easy to see that the interpretation we define above is a model of CORE. For the rest of the paper, we slightly abuse notation by calling CORE both the theory and its model making distinctions when necessary.

*Remark 1.* It is worth noticing the *syntactic* character of CORE: by interpreting atoms as themselves and since there are no equational axioms, we easily connect $\nabla \models a\#t$ and $\nabla \vdash a\#t$. This behaviour is not the rule if equational axioms are considered. For instance, consider the theory LAM that axiomatises $\beta$-equality in the $\lambda$-calculus. It is a fact that $a\#_{\mathsf{sem}}\overline{(\lambda[a]b)a}$ in LAM but there is no syntactic derivation for $a\#(\lambda[a]b)a$. Furthermore, by completeness for equality derivation, we establish a connection between $\nabla \models s \approx_\alpha t$ and $\nabla \vdash s \approx_\alpha t$.

There are alternative definitions of nominal terms where the syntax is many-sorted. We chose to work with an unsorted syntax for simplicity; all the results below can be extended to the many-sorted case, indeed they are proved for any infinite subalgebra of the ground nominal algebra.

### 3 Nominal Equational Problems

In this section, we introduce *nominal equational problems* (`NEP`s) as our main object of study. A `NEP` is a fist-order formula built only with the predicates $\approx_\alpha$

and $\#$. Their negations, denoted $\not\approx_\alpha$ and $\#\!\!\!\!\setminus$, are used to build disequations and non-freshness constraints. A *trivial disequation* is either $s \not\approx_\alpha s$ or $\perp$.

Intuitively, a non-freshness constraint $a\#\!\!\!\!\setminus t$ — read *a is not fresh for t* — states that there exists at least one instance of $t$ where $a$ occurs free. Similarly, for disequations: $s \not\approx_\alpha t$ states that $s$ and $t$ are not $\alpha$-equivalent.

**Definition 4.** *A* nominal system *is a formula defined by the following grammar:*

$$P, P' ::= \top \mid \perp \mid s \approx_\alpha t \mid s \not\approx_\alpha t \mid a\#t \mid a\#\!\!\!\!\setminus t \mid P \wedge P' \mid P \vee P'$$

In the next definition, we make a distinction between the set of variables occurring in a `NEP`: the mutually disjoint sets $\overline{W} = \{W_1, \ldots, W_n\}$ and $\overline{Y} = \{Y_1, \ldots, Y_m\}$ denote existentially and universally quantified variables, respectively. The former we call *auxiliary variables* and the latter *parameters.*

**Definition 5 (`NEP`).** *A* `NEP` *is a formula of the form below, where $P$ is a nominal system.*

$$\mathcal{P} ::= \exists W_1 \ldots W_n \forall Y_1 \ldots Y_m : P$$

The set $\mathtt{Fv}(\mathcal{P})$ contains the free variables occurring in $\mathcal{P}$. For the rest of the paper, we use the following implicit naming scheme for variables: $W$ denotes an auxiliary variable, $Y$ a parameter, $X$ a free variable, and $Z$ an arbitrary variable.

*Example 2.* Nominal disunification constraints [1] are pairs of the form $\mathcal{P} := \exists\overline{W}\langle E \mid\mid D\rangle$, where $E$ is a finite set of nominal equations-in-context, i.e., $E = \bigcup_{i=0}^{n}\{\Delta_i \vdash s_i \approx_\alpha t_i\}$ and $D$ is a finite set of nominal disequations-in-context, $D = \bigcup_{j=0}^{m}\{\nabla_j \vdash u_j \not\approx_\alpha v_j\}$. This problem is a particular `NEP`: taking the judgement $\Delta \vdash s \approx_\alpha t$ as $\Delta \Rightarrow s \approx_\alpha t$, or yet as $\neg\Delta \vee s \approx_\alpha t$[4], we obtain the formula:

$$\mathcal{P} := (\bigwedge_{i=0}^{n}(\neg[\Delta_i] \vee s_i \approx_\alpha t_i)) \wedge (\bigwedge_{j=0}^{m}(\neg[\nabla_j] \vee u_j \not\approx_\alpha v_j)),$$

where $[\Delta_i], [\nabla_j]$ are conjunctions of freshness constraints in $\Delta_i$, $\nabla_j$, respectively.

*Sufficient completeness*, that is, deciding whether a set of pattern (rules) covers all possible cases, is a well-known problem in functional programming. In the next example, we show how to naturally represent such problems as `NEPs`.

*Example 3.* Consider the function $\mathsf{map}$ which applies a function $[a]F$ to every element of any list $L$. It may be defined by the rules below:

$$\mathcal{R}_{\mathsf{map}} = \begin{cases} \vdash \mathsf{map}([a]F, \mathsf{nil}) \to \mathsf{nil} \\ \vdash \mathsf{map}([a]F, \mathsf{cons}(X, L)) \to \mathsf{cons}(F\{a \mapsto X\}, \mathsf{map}([a]F, L)), \end{cases}$$

[4] Similarly, for disequations.

where $\_\{a \mapsto \_\}$ is a binary term-former representing (explicit) substitutions; see [14, Example 43] for more details. Since we are not imposing a type discipline on nominal terms it is possible to construct ill-typed terms, for instance $\mathsf{map}(a, [a]t)$. In what follows we ignore those expressions by noticing that a type discipline will not allow such constructions. Then sufficient completeness can be checked using the following NEP:

$$\forall Y_1 Y_2 Y_3 L' : \mathsf{map}([a]F, L) \not\approx_\alpha \mathsf{map}([b]Y_1, \mathsf{nil}) \wedge \\ \mathsf{map}([a]F, L) \not\approx_\alpha \mathsf{map}([b]Y_2, \mathsf{cons}(Y_3, L')),$$

If the problem has a solution then $\mathcal{R}_{\mathsf{map}}$ is not complete, and the solution indicates the missing pattern cases in the definition.

*Solutions of Nominal Equational Problems.* We are interested in solutions for NEPs in the ground nominal algebra. From now on, $\mathcal{A}$ denotes an infinite subalgebra of CORE with domain $A$. Below we define solutions using idempotent substitutions, which can be seen as a representation for valuations that map variables to elements of the ground term algebra.

We first extend the interpretation function under a valuation $\varsigma$ $[\![\cdot]\!]_\varsigma$ (see Section 2) to the negated form of freshness and $\alpha$-equality constraints.

**Definition 6.** *Let $\varsigma$ be a (fixed but arbitrarily given) valuation. A negative constraint $a \not\#\, t$ (resp. $s \not\approx_\alpha t$) is valid under $\varsigma$ when:*

- *it is not the case that $a \#_{\mathsf{sem}} [\![t]\!]_\varsigma$, this is written $[\![a \not\#\, t]\!]_\varsigma$; and, respectively,*
- *it is not the case that $[\![s]\!]_\varsigma = [\![t]\!]_\varsigma$, this is written $[\![s \not\approx_\alpha t]\!]_\varsigma$.*

In standard unification algorithms, idempotent substitutions are used as a compact representation of a set of valuations in the ground term algebra. Similarly, given a valuation in the ground term algebra, one can build a ground substitution representing it. In the case of the ground nominal algebra, where elements are $\alpha$-equivalence classes of terms, the representative is generally not unique, but any representative can be used.

**Definition 7.** *Given a substitution $\sigma = [X_1/t_1, \ldots, X_n/t_n]$, for any valuation $\varsigma$, we denote by $\varsigma^\sigma$ the valuation such that $\varsigma^\sigma(X) = \varsigma(X)$ if $X \notin \mathtt{dom}(\sigma)$, and $\varsigma^\sigma(X) = [\![X\sigma]\!]_\varsigma$ otherwise.*

*Given a valuation $\varsigma = [X_i \mapsto g_i \mid X_i \in \mathbb{X}, g_i \in A]$, and a finite set $\mathcal{X}$ of variables, we denote by $\sigma^\varsigma_\mathcal{X}$ any ground substitution such that for each $X_i \in \mathcal{X}$, $\sigma(X_i) = t_i$, if $g_i = [\![t_i]\!]_\varsigma$. We say that $\sigma^\varsigma_\mathcal{X}$ is a grounding substitution for $\mathcal{X}$.*

The next lemma states that under mild conditions we can extend substitutions to valuations preserving semantic equality.

**Lemma 1.** *Given an idempotent substitution $\sigma = [X_1/t_1, \ldots, X_n/t_n]$ and a valuation $\varsigma$ we have: $[\![s\sigma]\!]_\varsigma = [\![s]\!]_{\varsigma^\sigma}$.*

The next definition allows us to use idempotent substitutions to represent solutions of constraints.

**Definition 8 (Constraint $\mathcal{A}$-validation).** *Let $\sigma$ be an idempotent substitution whose domain includes all the variables occurring in a constraint $C$. Then $\sigma$ $\mathcal{A}$-validates $C$ iff $[\![C]\!]_{\varsigma^\sigma}$ is valid in $\mathcal{A}$ for any valuation $\varsigma$.*

We now extend semantic validity to the syntax of systems. The interpretation for the logical connectives is defined as expected.

**Definition 9 ($\mathcal{A}$-validation).** *For an idempotent substitution $\sigma$ whose domain includes all variables occurring in a system $P$, we say that $\sigma$ $\mathcal{A}$*-validates *$P$ iff*

1. *$P = \top$; or*
2. *$P = C$ and $\sigma$ $\mathcal{A}$-validates $C$; or*
3. *$P = P_1 \wedge \ldots \wedge P_n$ and $\sigma$ $\mathcal{A}$-validates each $P_i$, $1 \leq i \leq n$; or*
4. *$P = P_1 \vee \ldots \vee P_m$ and $\sigma$ $\mathcal{A}$-validates at least one $P_i$, $1 \leq i \leq m$.*

Solutions of equational problems instantiate free variables and satisfy existential and universal requirements for auxiliary variables and parameters, respectively. To define this notion, we extend the domain of the substitution to include also existential and universally quantified variables as follows.

**Definition 10 ($\mathcal{A}$-Solution).** *Let $\mathcal{P} = \exists\overline{W}\forall\overline{Y} : P$ be a NEP. Let $\sigma$ be an idempotent substitution such that $\mathtt{dom}(\sigma) = \mathit{Fv}(\mathcal{P})$. Then $\sigma$ is an $\mathcal{A}$*-solution *of $\mathcal{P}$ iff there is a ground substitution $\delta$, where $\mathtt{dom}(\delta) = \overline{W}$, such that for all ground substitution $\lambda$, where $\mathtt{dom}(\lambda) = \overline{Y}$, $\sigma\delta\lambda$ $\mathcal{A}$-validates $P$. The set of $\mathcal{A}$-solutions of $\mathcal{P}$ is denoted $\mathcal{S}_{\mathcal{A}}(\mathcal{P})$, or simply $\mathcal{S}(\mathcal{P})$ if $\mathcal{A}$ is clear from the context.*

*Example 4.* Consider the signature $\Sigma_{\mathsf{nat}} := \{\mathsf{zero} : 0, \mathsf{suc} : 1\}$ for natural numbers, and the nominal initial algebra $\mathcal{A}_{\mathsf{nat}}$ with $\mathsf{zero}$ and $\mathsf{suc}$ interpreted as expected. The problem $\mathcal{P} := \exists W \forall Y : W \not\approx_\alpha \mathsf{suc}(Y)$ has $\mathtt{id}$ as solution. Indeed, taking for example $\delta = [W/\mathsf{zero}]$ or $\delta = [W/a]$ and any choice of $\lambda$ ($\mathtt{dom}(\lambda) = \{Y\}$), the composition $\mathtt{id}\delta\lambda$ $\mathcal{A}$-validates $W \not\approx_\alpha \mathsf{suc}(Y)$.

In Definition 10, $\delta$ is the substitution that instantiates auxiliary variables, so there can be many (possibly infinite) number of such $\delta$'s.

**Lemma 2 (Equivariance of Solutions).** *If $\sigma$ is an $\mathcal{A}$*-solution *of the NEP $\mathcal{P}$ then for any permutation $\pi$, $\pi \cdot \sigma$ (defined by $[X_i/\pi \cdot t_i]$, as expected) is an $\mathcal{A}$*-solution *of $\pi \cdot \mathcal{P}$. In particular, if an $\mathcal{A}$*-solution *contains an atom not occurring in $\mathcal{P}$, that atom can be swapped for any other atom not occurring in $\mathcal{P}$.*

Lemma 2 is a direct consequence of the fact that interpretations are equivariant, and shows that solutions are closed by permutation. It allows us to use permutations to represent infinite choices for atoms in solutions.

*Example 5.* Consider the problem $\forall Y : X \not\approx_\alpha \lambda[a]Y$, built over the signature of Example 1. The set of solutions contains $\sigma = [X/a]$ as well as $(a\ b) \cdot [X/a] = [X/b]$; for any other atom $b$.

**Lemma 3 (Closure by Instantiation).** *If $\sigma$ is an $\mathcal{A}$-solution of the* NEP $\mathcal{P} = \exists\overline{W}\forall\overline{Y} : P$ *then any idempotent substitution $\sigma'$ obtained as an instance of $\sigma$ such that* $\mathtt{dom}(\sigma') = \mathtt{dom}(\sigma)$ *is also an $\mathcal{A}$-solution of $\mathcal{P}$. In particular, for any such ground instance $\sigma'$ of $\sigma$ there is a ground substitution $\delta$, where* $\mathtt{dom}(\delta) = \overline{W}$*, such that for all ground substitution $\lambda$, where* $\mathtt{dom}(\lambda) = \overline{Y}$*, $\sigma'\delta\lambda$ $\mathcal{A}$-validates $P$.*

*Proof.* By definition of $\mathcal{A}$-solution, to show that $\sigma'$ is an $\mathcal{A}$-solution of $\mathcal{P}$ we need to consider all the valuations of the form $\varsigma^{\sigma'\delta\lambda}$ as indicated in Definitions 8, 9, 10. The result follows from the fact that for any valuation $\varsigma^{\sigma'\delta\lambda}$ there exists an equivalent valuation $\varsigma'^{\sigma\delta\lambda}$ by Lemma 1.

## 4 A rule-based procedure

In this section we present a set of simplification rules to solve NEPs. A simplification step, denoted $\mathcal{P} \Longrightarrow \mathcal{P}'$, transforms $\mathcal{P}$ into an equivalent problem $\mathcal{P}'$ from which solutions are easier to extract.

### 4.1 Simplification Rules

Rules may have application conditions (rule controls) that define a strategy of simplification. Our strategy gives priority to rules according to their role. We split the rules into groups $\mathcal{R}_i$ as shown in Figures 2, 3 and 4: $\mathcal{R}_1$ eliminates trivial constraints, $\mathcal{R}_2$ deals with clash and occurs check, $\mathcal{R}_3$ eliminates unneeded quantifiers, $\mathcal{R}_4$ and $\mathcal{R}_5$ decompose positive and negative constraints, respectively, $\mathcal{R}_6$ eliminates parameters and $\mathcal{R}_7$ instantiates variables. The Explosion and Elimination of Disjunction rules in $\mathcal{R}_8$ search for solutions as explained below. Finally, $\mathcal{R}_9$ eliminates the remaining universal quantifiers. A rule $R \in \mathcal{R}_i$ can only be applied if no rules from $\mathcal{R}_j$, where $j < i$, can be applied.

Since we are dealing with formulas that contain disjunction and conjunction connectives, we need to take into account the standard Boolean axioms. To simplify, instead of working modulo the Boolean axioms we apply a Boolean normalisation step before a rule is applied. Following Comon and Lescanne [10], we choose to take *conjunctive normal form*: Before the application of each rule $\mathcal{P}$ is reduced to a conjunction of disjunctions.

The explosion rule creates new branches by instantiating variables considering all possible ways of constructing terms (i.e., each $f \in \Sigma$, abstractions and atoms). Note that $\Sigma \cup \mathtt{Atoms}(P) \cup \{a'\}$ is a finite set (we can represent all possible constructions with a finite number of cases), so the rule is finitely branching.

The rule Elimination of Disjunctions also builds a finite number of branches. Therefore, our procedure builds a finitely branching tree of problems to be solved.

Rules $\mathcal{R}_1$-$\mathcal{R}_8$ are not sufficient to eliminate all parameters from a NEP (see Example 6) in contrast with the syntactic case [7], where similar rules produce parameterless normal forms. This is because we are dealing with both freshness and $\alpha$-equality. Indeed, normal forms for rules $\mathcal{R}_1$-$\mathcal{R}_8$ may contain parameters, but only in disjunctions involving both freshness and equality constraints for the same parameter as the following lemma states. The rules in $\mathcal{R}_9$ (Figure 4) are introduced to deal with this problem.

$\mathcal{R}_1$ : Trivial Rules

| | | |
|---|---|---|
| $(T_1)$ $t \approx_\alpha t \Longrightarrow \top$ | $(T_2)$ $t \not\approx_\alpha t \Longrightarrow \bot$ | $(T_3)$ $a \approx_\alpha b \Longrightarrow \bot$ |
| $(T_4)$ $a\#b \Longrightarrow \top$ | $(T_5)$ $a\#a \Longrightarrow \bot$ | $(T_6)$ $a\not\#a \Longrightarrow \top$ |
| $(T_7)$ $a\not\#b \Longrightarrow \bot$ | $(T_8)$ $a\#t \wedge a\not\#t \Longrightarrow \bot$ | $(T_9)$ $a\#t \vee a\not\#t \Longrightarrow \top$ |

$\mathcal{R}_2$: Clash and Occurrence Check Rules

$(CL_1)$ $s \not\approx_\alpha t \Longrightarrow \top$ $(CL_2)$ $s \approx_\alpha t \Longrightarrow \bot$

**Conditions for** $(CL_1)$ **and** $(CL_2)$**:** $s(\epsilon) \neq t(\epsilon)$ and neither is a moderated variable.

$(O_1)$ $\pi \cdot Z \approx_\alpha t \Longrightarrow \bot$ $(O_2)$ $\pi \cdot Z \not\approx_\alpha t \Longrightarrow \top$

**Conditions for** $(O_1)$ **and** $(O_2)$**:** $Z \in \mathtt{vars}(t)$ and $t \not\equiv \pi' \cdot Z$

$\mathcal{R}_3$: Elimination of parameters and auxiliary unknowns.

| | |
|---|---|
| $(C_1)$ $\forall\overline{Y}, Y : P \Longrightarrow \forall\overline{Y} : P,$ | $Y \notin \mathtt{vars}(P)$ |
| $(C_2)$ $\exists\overline{W}, W : P \Longrightarrow \exists\overline{W} : P,$ | $W \notin \mathtt{vars}(P)$ |
| $(C_3)$ $\exists\overline{W}, W : \pi \cdot W \approx_\alpha t \wedge P \Longrightarrow \exists\overline{W} : P,$ | $W \notin \mathtt{vars}(P, t)$ |

$\mathcal{R}_4$: Equality and freshness simplification

| | | | |
|---|---|---|---|
| $(E_1)$ | $\pi \cdot X \approx_\alpha \gamma \cdot X \Longrightarrow \wedge\, \mathtt{ds}(\pi, \gamma)\#X$ | $(F_1)$ | $a\#\pi \cdot X \Longrightarrow \pi^{-1}(a)\#X, \pi \neq \mathtt{id}$ |
| $(E_2)$ | $[a]t \approx_\alpha [a]u \Longrightarrow t \approx_\alpha u$ | $(F_2)$ | $a\#[a]t \Longrightarrow \top$ |
| $(E_3)$ | $[a]t \approx_\alpha [b]u \Longrightarrow (b\ a) \cdot t \approx_\alpha u \wedge b\#t$ | $(F_3)$ | $a\#[b]t \Longrightarrow a\#t$ |
| $(E_4)$ | $f(\tilde{t}) \approx_\alpha f(\tilde{u}) \Longrightarrow \wedge_i\, t_i \approx_\alpha u_i$ | $(F_4)$ | $a\#f(t_1, \ldots, t_n) \Longrightarrow \wedge_i a\#t_i$ |

$\mathcal{R}_5$: Disunification

| | | | |
|---|---|---|---|
| $(DC)$ | $f(\tilde{t}) \not\approx_\alpha f(\tilde{u}) \Longrightarrow \vee_i\, t_i \not\approx_\alpha u_i$ | $(NF_1)$ | $a\not\#\pi \cdot X \Longrightarrow \pi^{-1}(a)\not\#X, \pi \neq \mathtt{id}$ |
| $(D_1)$ | $\pi \cdot X \not\approx_\alpha \gamma \cdot X \Longrightarrow \vee_i\, \mathtt{ds}(\pi, \gamma)\not\#X$ | $(NF_2)$ | $a\not\#[a]t \Longrightarrow \bot$ |
| $(D_2)$ | $[a]t \not\approx_\alpha [a]u \Longrightarrow t \not\approx_\alpha u$ | $(NF_3)$ | $a\not\#[b]t \Longrightarrow a\not\#t$ |
| $(D_3)$ | $[a]t \not\approx_\alpha [b]u \Longrightarrow (b\ a) \cdot t \not\approx_\alpha u \vee b\not\#t$ | $(NF_4)$ | $a\not\#f(\tilde{t}) \Longrightarrow \vee_i a\not\#t_i$ |

$\mathcal{R}_6$: Simplification of Parameters

$(U_1)$ $\forall\overline{Y}, Y : P \wedge \pi \cdot Y \not\approx_\alpha t \Longrightarrow \bot$ if $Y \notin \mathtt{vars}(t)$

$(U_2)$ $\forall\overline{Y} : P \wedge (\pi \cdot Y \not\approx_\alpha t \vee Q) \Longrightarrow \forall\overline{Y} : P \wedge Q[Y/\pi^{-1} \cdot t]$, if $Y \notin \mathtt{vars}(t)$, $Y \in \overline{Y}$

$(U_3)$ $\forall\overline{Y}, Y : P \wedge \pi \cdot Y \approx_\alpha t \Longrightarrow \bot$, if $\pi \cdot Y \not\equiv t$

$(U_4)$ $\forall\overline{Y} : P \wedge (\pi_1 \cdot Z_1 \approx_\alpha t_1 \vee \cdots \vee \pi_n \cdot Z_n \approx_\alpha t_n \vee Q) \Longrightarrow \forall\overline{Y} : P \wedge Q$

$(U_5)$ $\forall\overline{Y}, Y : P \wedge a\#Y \Longrightarrow \bot$

$(U_6)$ $\forall\overline{Y}, Y : P \wedge a\not\#Y \Longrightarrow \bot$

**Conditions for** $(U_4)$**:**

- Each equation in the disjunction contains at least one occurrence of a parameter and $\pi_i \cdot Z_i \not\equiv t_i$ for each $i = 1, \ldots, n$.
- $Q$ does not contain any parameter.

$\mathcal{R}_7$: Instantiation Rules

$(I_1)$ $\pi \cdot Z \approx_\alpha t \wedge P \Longrightarrow Z \approx_\alpha \pi^{-1} \cdot t \wedge P[Z/\pi^{-1} \cdot t]$

- If $\pi = \mathtt{id}$ then $Z$ is not a parameter and $Z$ occurs in $P$ and if $t$ is a variable then $t$ occurs in $P$.
- If $\pi \neq \mathtt{id}$, then $t$ is not of the form $\mathtt{id} \cdot Z'$.

$(I_2)$ $\pi \cdot Z \not\approx_\alpha t \vee P \Longrightarrow Z \not\approx_\alpha \pi^{-1} \cdot t \vee P[Z/\pi^{-1} \cdot t]$

- If $\pi = \mathtt{id}$ then $Z$ occurs in $P$ and if $t$ is a variable then $t$ occurs in $P$.
- If $\pi \neq \mathtt{id}$ then $t$ is not of the form $\mathtt{id} \cdot Z'$.

**Fig. 2.** Preserving Rules

$\mathcal{R}_8$: Explosion and Elimination of Disjunction

$(ED_1)\, \forall\overline{Y} : P \wedge (P_1 \vee P_2) \Longrightarrow \forall\overline{Y} : P \wedge P_1$, if $\mathtt{vars}(P_1) \cap \overline{Y} = \emptyset$ or $\mathtt{vars}(P_2) \cap \overline{Y} = \emptyset$.

$(ED_2)\ \forall\overline{Y_1}, \overline{Y_2} : P \wedge (P_1 \vee P_2) \Longrightarrow \forall\overline{Y_1}, \overline{Y_2} : P \wedge P_1$, if $\mathtt{vars}(P_1) \cap \overline{Y_2} = \emptyset$ and $\mathtt{vars}(P_2) \cap \overline{Y_1} = \emptyset$

$(\mathit{Exp})\ \exists\overline{W}\forall\overline{Y} : P \Longrightarrow \exists\overline{W'}\exists\overline{W}\forall\overline{Y} : P \wedge X \approx_\alpha t$, for $t = f(\overline{W'})$ or $t = [a]W'$ or $t = a$

**Conditions for** $(\mathit{Exp})$**:**

1. $X$ is a free or existential variable occurring in $P$, $\overline{W'}$ are newly chosen auxiliary variables not occurring anywhere in the problem;
2. $f \in \Sigma$ and $a \in \mathtt{Atoms}(P) \cup \{a'\}$, where $a'$ is a new atom;
3. there exists an equation $X \approx_\alpha u$ (or disequation $X \not\approx_\alpha u$) in $P$ such that $u$ is not a variable and contains at least one parameter; and
4. no other rule can be applied.

**Fig. 3.** Globally Preserving Rules

*Example 6.* Both $\mathcal{P} = a\#Y_1 \vee Y \approx_\alpha f(Y_1)$ and $\mathcal{P} = a\#Y_1 \vee a\not\#Y \vee Y_1 \approx_\alpha f(Y)$ are irreducible: neither $(U_4)$ nor $(ED_1)$ apply since all the disjuncts contain parameters; $(ED_2)$ does not apply since each constraint has a parameter that occurs in another constraint; $(\mathit{Exp})$ does not apply because there is no equation or disequation with a free or existentially quantified variable in one side.

The following lemma characterises the irreducible disjunctions with respect to rules $\mathcal{R}_1$-$\mathcal{R}_8$ where parameters may remain.

**Lemma 4.** *Let $P$ be a disjunction of constraints irreducible w.r.t. $\mathcal{R}_1$-$\mathcal{R}_8$. For each parameter $Y$ such that $P = a\#Y \vee Q$ (resp. $P = a\not\#Y \vee Q$), for some atom $a$, the following holds:*

1. *$a\not\#Y$ (resp. $a\#Y$) cannot occur in $Q$;*
2. *$Y$ has to occur in $Q$;*
3. *if $Q$ contains an equational constraint then it has the form $Y \approx_\alpha t$, where $Y \notin \mathtt{vars}(t)$, or $Y' \approx_\alpha t$, with $Y \in \mathtt{vars}(t)$;*
4. *$Q$ does not contain disequations or primitive freshness constraints for free or existentially quantified variables.*

*Proof.* In an irreducible disjunction of constraints at least one of the sides of equations (or disequations) is a variable, otherwise we could simplify the equation/disequation.

**Condition 1.** It holds, otherwise we could apply $(T_9)$. **Condition 2.** It holds, otherwise we could apply $(ED_2)$.

**Condition 3.** If $Q$ had an equation of the form $X \approx_\alpha t$, for some free or existentially quantified variable, then $t$ could not contain a parameter, otherwise we could apply rule (Exp). Therefore, $t = t[Z_1, \ldots, Z_n]$, for $n \geq 0$ where each $Z_i$

| $\mathcal{R}_9$: Simplification of parameters in freshness constraints |
|---|
| $(U_7)$ $\forall \overline{Y}, Y : P \wedge (a\#Y \vee Q) \Longrightarrow \bot$ |
| if $\mathcal{R}_1$-$\mathcal{R}_8$ do not apply (so $Q$ does not contain $a\not\#Y$) and $Y \in \mathtt{vars}(Q)$. |
| $(U_8)$ $\forall \overline{Y}, Y : P \wedge (a\not\#Y \vee Q) \Longrightarrow \bot$ |
| if $\mathcal{R}_1$-$\mathcal{R}_8$ do not apply (so $Q$ does not contain $a\#Y$) and $Y \in \mathtt{vars}(Q)$. |

**Fig. 4.** Preserving Rules for (non)freshness constraints with parameters.

is either a free or existentially quantified variable, and one could apply rule $ED_1$. Thus, if an equation exists, one of the sides has to be a parameter, say $Y \approx_\alpha t$, and $Y$ cannot occur in $t$ otherwise rule $O_2$ applies.

**Condition 4.** If $Q$ were to contain a disequation, say $X \not\approx_\alpha t$ then $t$ could not contain a parameter, otherwise we could apply (Exp) as above, but then we could apply rule $(ED_1)$. Therefore, if $Q$ were to contain a disequation, it would be of the form $Y \not\approx_\alpha t$, then it would either reduce with $(O_2)$ or with $(U_2)$. Thus, $Q$ does not contain disequations. Similary, if $Q$ contained a primitive freshness constraint for a free or existentially quantified variable then $(ED_1)$ would apply.

The remaining disjunctions with parameters can be simplified using the rules in $\mathcal{R}_9$, since they will not produce solutions (as shown in Theorem 1).

We end this section with an example of application of the simplification rules.

*Example 7.* Let $\mathcal{P}$ be a `NEP`, using the signature from Example 1, as follows:

$$\mathcal{P} = \forall Y : \lambda[a]X \not\approx_\alpha \lambda[a]\lambda[a]Y \overset{DC}{\Longrightarrow} \forall Y : [a]X \not\approx_\alpha [a]\lambda[a]Y \overset{D_2}{\Longrightarrow} \forall Y : X \not\approx_\alpha \lambda[a]Y$$

Rules in $\mathcal{R}_1$-$\mathcal{R}_7$ cannot be applied and the explosion rule produces six problems:

$$\begin{array}{ll} \mathcal{P}_1 = \exists W_1 \forall Y : X \not\approx_\alpha \lambda[a]Y \wedge X \approx_\alpha \lambda W_1 & \mathcal{P}_4 = \exists W \forall Y : X \not\approx_\alpha [a]Y \wedge X = [b]W \\ \mathcal{P}_2 = \exists W_1, W_2 \forall Y : X \not\approx_\alpha [a]Y \wedge X = W_1 W_2 & \mathcal{P}_5 = \exists W \forall Y : X \not\approx_\alpha [a]Y \wedge X = a \\ \mathcal{P}_3 = \exists W \forall Y : X \not\approx_\alpha [a]Y \wedge X = [a]W & \mathcal{P}_6 = \forall Y : X \not\approx_\alpha [a]Y \wedge X = b \end{array}$$

Reducing the first problem we get:

$$\begin{aligned} \mathcal{P}_1 &\overset{I_1}{\Longrightarrow} \exists W_1 \forall Y : \lambda W_1 \not\approx_\alpha \lambda[a]Y \wedge X \approx_\alpha \lambda W_1 \\ &\overset{DC}{\Longrightarrow} \exists W_1 \forall Y : W_1 \not\approx_\alpha [a]Y \wedge X \approx_\alpha \lambda W_1 \\ &\overset{Exp}{\Longrightarrow} \exists W_1 W_2 \forall Y : W_1 \not\approx_\alpha [a]Y \wedge X \approx_\alpha \lambda W_1 \wedge W_1 \approx_\alpha \lambda W_2 \\ &\overset{I_1}{\Longrightarrow} \exists W_1 W_2 \forall Y : \lambda W_2 \not\approx_\alpha [a]Y \wedge X \approx_\alpha \lambda W_1 \wedge W_1 \approx_\alpha \lambda W_2 \\ &\overset{CL_1}{\Longrightarrow} \exists W_1 W_2 \forall Y : X \approx_\alpha \lambda W_1 \wedge W_1 \approx_\alpha \lambda W_2 \\ &\overset{I_1}{\Longrightarrow} \exists W_1 W_2 : X \approx_\alpha \lambda\lambda W_2 \wedge W_1 \approx_\alpha \lambda W_2. \end{aligned}$$

At this point $\mathcal{P}_1$ has reached a normal form without any parameter. Solutions of $\mathcal{P}_1$ can be easily obtained by taking any instance of $X$ of the form $\lambda\lambda t$. It is easy to check that this choice indeed generates solutions of $\mathcal{P}$. Similar reductions apply to $\mathcal{P}_i$, $2 \leq i \leq 6$.

As we will see in the next section, application of such simplification rules is *well-behaved* in the sense that we do not loose any solution along the way.

#### 4.2 Soundness and Preservation of Solutions

The next step is to ensure that the application of rules does not change the set of solutions of an equational problem.

**Definition 11 (Soundness and preservation of solution).** *Let $\mathcal{A}$ be any infinite subalgebra of* CORE.

1. *A rule $R$ is $\mathcal{A}$-*sound *if, $\mathcal{P} \Longrightarrow_R \mathcal{P}'$ implies $\mathcal{S}(\mathcal{P}') \subseteq \mathcal{S}(\mathcal{P})$.*
2. *A rule $R$ is $\mathcal{A}$-*preserving *if, $\mathcal{P} \Longrightarrow_{\mathcal{R}} \mathcal{P}'$ implies $\mathcal{S}(\mathcal{P}) \subseteq \mathcal{S}(\mathcal{P}')$.*
3. *A rule $R$ is $\mathcal{A}$-*globally preserving *if given any problem $\mathcal{P}$,*

$$\mathcal{S}(\mathcal{P}) \subseteq \bigcup_{\substack{\mathcal{P} \to_{\mathcal{R}} \pi \cdot \mathcal{P}_i \\ \mathtt{supp}(\pi) \cap \mathtt{Atoms}(\mathcal{P}) = \emptyset}} \mathcal{S}(\mathcal{P}_i).$$

All our rules, except those in $\mathcal{R}_8$, are sound and preserving (Theorem 1). The rules in $\mathcal{R}_8$ create branches in the derivation tree; they are sound and only globally preserving (Theorem 2).

**Theorem 1.** *The rules in $\mathcal{R}_1$ to $\mathcal{R}_7$ and the rules in $\mathcal{R}_9$ are $\mathcal{A}$-sound and $\mathcal{A}$-preserving for any infinite subalgebra $\mathcal{A}$ of* CORE.

*Proof.* **Rules in $\boldsymbol{\mathcal{R}}_1$, $\boldsymbol{\mathcal{R}}_2$, and $\boldsymbol{\mathcal{R}}_3$** : soundness and preservation of solutions are easy to deduce. For instance, for clash rules, $(CL_1)$ and $(CL_2)$, it follows by inspection of deduction rules that the judgement $\vdash s\gamma \approx_\alpha t\gamma$ is not derivable for any valuation $\varsigma$ and corresponding grounding substitution $\gamma = \sigma^{\varsigma}_{\mathtt{vars}(s,t)}$ (see Definition 7) if the root constructors of $s$ and $t$ are different (hence every $\gamma$ is a solution for the disequation). For $(C_3)$ observe that we can take $[W/t]$ as a witness for $W$ on a validation for $\exists \overline{W} : P$, if $W \notin \mathtt{vars}(P, t)$.
**Rules in $\boldsymbol{\mathcal{R}}_4$** and **$\boldsymbol{\mathcal{R}}_5$**. It follows from soundness and preservation of simplification rules in [14]. We use the fact that nominal equality and freshness rules from Fig. 1 are reversible; for instance, let $\gamma$ be a grounding substitution, a judgement $\vdash f(\tilde{s})\gamma \approx_\alpha f(\tilde{u})\gamma$ fails, which makes $f(\tilde{s})\gamma \not\approx_\alpha f(\tilde{u})\gamma$ valid, iff one of the premises $\vdash s_i\gamma \approx_\alpha u_i\gamma$ does not hold.
**Rules in $\boldsymbol{\mathcal{R}}_6$**: The result is straightforward for rules $U_1$ and $U_3$.

$\boldsymbol{U_2}$. To prove soundness for $U_2$ notice that the solution set of a conjunction is the intersection of the solution set of each of its members. We have to show that every solution of $Q[Y/\pi^{-1} \cdot t]$ is a solution of $(\pi \cdot Y \not\approx_\alpha t \vee Q)$. Let $\gamma$ be a solution of $Q[Y/\pi^{-1} \cdot t]$ and take any substitution $\lambda$ satisfying the conditions of Definition 10. So $(Q[Y/\pi^{-1} \cdot t])\gamma\lambda$ is valid and we need to show the validity of

$$(\pi \cdot Y \not\approx_\alpha t)\gamma\lambda \vee Q\gamma\lambda. \tag{1}$$

For each such $\lambda$ there are two possible cases: First, $\vdash \pi \cdot Y\lambda \approx_\alpha t\gamma\lambda$ (note that $\lambda$ is a ground substitution so both sides of this equation are ground); then we have that $\gamma\lambda = \gamma\lambda'[Y/\pi^{-1} \cdot t\gamma\lambda]$. By hypothesis, $\gamma\lambda$ validates $Q[Y/\pi^{-1} \cdot t]$

so $\gamma\lambda'[Y/\pi^{-1} \cdot t\gamma\lambda]$ validates $Q$. Second; $\not\vdash \pi \cdot Y\lambda \approx_\alpha t\gamma\lambda$, then $\gamma\lambda$ validates $\pi \cdot Y \not\approx_\alpha t$. Hence $\gamma$ a solution of (1).

To prove preservation for $\boldsymbol{U}_2$, take $\gamma$ a solution of $\forall\overline{Y}, Y : \pi \cdot Y \not\approx_\alpha t \vee Q$, we need to show that $\gamma$ is also a solution of $\forall\overline{Y}, Y : Q[Y/\pi^{-1} \cdot t]$. Notice that $\gamma$ is a solution of $\forall\overline{Y}, Y : \pi \cdot Y \not\approx_\alpha t$ or $\forall\overline{Y}, Y : Q$ but it clearly cannot solve the first problem. Hence, $\gamma$ solves $\forall\overline{Y}, Y : Q$. By Definition 10, for all substitutions $\lambda$ with domain $\overline{Y} \cup \{Y\}$ we have that $\lambda\gamma$ validates $Q$. In particular, the substitution $[Y/\pi^{-1} \cdot t\gamma]\lambda\gamma$ which is equivalent to $[Y/\pi^{-1} \cdot t]\lambda\gamma$ (since $\gamma$ is away from $\lambda$) must also validate $Q$. Consequently, $\lambda\gamma$ validates $(Q[Y/\pi^{-1} \cdot t])$.

$\boldsymbol{U}_4$. Soundness for this rule follows trivially. For preservation of solutions, we show that any solution of $\forall\overline{Y} : \bigvee_i Z_i \approx_\alpha t_i \vee Q$ is a solution of $\forall\overline{Y} : Q$. The shape of the first problem induces a requirement that the disjunction $\bigvee_i Z_i \approx_\alpha t_i$ does not have a solution. To show this we prove that the negated form $\bigwedge_i Z_i \not\approx_\alpha t_i$ has at least one solution. Notice that such a solution is a witness for the failure of $\bigvee_i Z_i \approx_\alpha t_i$, since all of those equations have at least one parameter. Lemma 5 shows that this is true.

$\boldsymbol{U}_5$ and $\boldsymbol{U}_6$. We need to show that every solution of $\forall\overline{Y}, Y : P \wedge a\#Y$ is also a solution of $\perp$, i.e., no such solution exists for the lhs of the rule. In fact, the existence of such $\gamma$ would imply that (taking $\lambda = [Y/a]$) $a\#a$ which is impossible. For $U_6$ we do the same reasoning with $\lambda = [Y/[a]a]$.

**Rules in $\boldsymbol{\mathcal{R}}_7$.** Soundness and preservation of $(I_1)$ has been proved in previous works, since rule $(I_1)$ is used in standard nominal unification algorithms [23]. Rule $(I_2)$ is a direct adaptation of the rule used in the standard (syntactic) case, proved sound and preserving in [10]. Indeed, $\gamma \in \mathcal{S}(\pi \cdot Z \not\approx_\alpha t \vee P)$ if, and only if, for any grounding instance $\gamma'$ of $\gamma$, $\gamma' \in \mathcal{S}(Z \not\approx_\alpha \pi^{-1} \cdot t)$ or $\gamma' \in \mathcal{S}(P)$ (by Lemma 3). Finally, notice that $\gamma \in \mathcal{S}(P) \setminus \mathcal{S}(Z \not\approx_\alpha \pi^{-1} \cdot t)$ if and only if $\gamma \in \mathcal{S}(P[Z/\pi^{-1} \cdot t])$.

**Rules in $\boldsymbol{\mathcal{R}}_9$.** Soundness follows trivially, since $\perp$ has no solution. We show below that $U_7$ is $\mathcal{A}$-preserving; the proof is analogous for rule $(U_8)$.

Let $\mathcal{P} = \exists\overline{W}\forall\overline{Y}, Y : P \wedge (a\#Y \vee Q)$ where $Q$ is fully reduced by $\mathcal{R}_1$-$\mathcal{R}_8$, $Y \in \mathtt{vars}(Q)$ and $Q$ does not contain $a\not\#Y$. We prove that $\mathcal{P}$ does not have solutions by induction on the number of freshness constraints in $a\#Y \vee Q$.

**Base case:** $Q$ contains just equational constraints, each containing at least one occurrence of the parameter $Y$, as specified in Lemma 4. Suppose by contradiction that there exists an $\mathcal{A}$-solution $\gamma$. Thus, $\gamma$ is away from $\overline{Y} \cup \{Y\}$, $\mathtt{dom}(\gamma) = \overline{X} = \mathtt{Fv}(\mathcal{P})$, there is a ground substitution $\delta$ with $\mathtt{dom}(\delta) = \overline{W}$ and for all $\lambda$ away from $\overline{X}, \overline{W}$, with $\mathtt{dom}(\lambda) = \overline{Y} \cup \{Y\}$, $\gamma\delta\lambda$ $\mathcal{A}$-validates $P \wedge (a\#Y \vee Q)$. Then, it $\mathcal{A}$-validates both $P$ and $(a\#Y \vee Q)$. The latter implies that $\gamma\delta\lambda$ $\mathcal{A}$-validates $Q$ for every $\lambda$ (but then $Q$ has a solution, which is impossible due to the form of the equational constraints) or $Q$ implies $a\not\#Y$ (since there is at least one $f \in \Sigma$ such that $f : n$ and $n > 0$, and therefore $a\#Y$ is false for an infinite number of ground terms $Y\lambda$). The latter is impossible since $a\#Y$ is defined as $a \notin \mathtt{supp}(Y)$, which is defined as $(a\,a') \cdot Y = Y$ for a new $a'$, and reduced problems cannot contain fixed point equations or their negations (these are simplified using rules $(E_1)$ and $(D_1)$, respectively).

The inductive step is proved similarly, using Lemma 4 as in the base case to deduce that the constraints in $Q$ cannot entail $a\not\#Y$.

**Theorem 2.** *Let $\mathcal{A}$ be any infinite subalgebra of* CORE*.*

1. *Rule (Exp) is $\mathcal{A}$-sound and $\mathcal{A}$-globally preserving.*
2. *Rules $(ED_1)$ and $(ED_2)$ are $\mathcal{A}$-sound and $\mathcal{A}$-globally preserving.*

Lemma 5 guarantees the existence of a solution for a conjunction of non-trivial disequations as long as the algebra considered has sufficient ground terms.

**Lemma 5.** *Let $\mathcal{P}$ be a conjunction of non-trivial disequations. Let $\mathcal{A}$ be any infinite subalgebra of* CORE*. Then $\mathcal{P}$ has at least one solution in $\mathcal{A}$.*

*Proof.* The proof proceeds by induction on the number of distinct variables occurring in $\mathcal{P}$. For the base case $\mathcal{P}$ has no variables. Then every substitution solves $\mathcal{P}$, since by hypothesis $\mathcal{P}$ does not contain any trivial disequation $t \not\approx_\alpha t$.

Assume the result holds for problems with $m-1$ variables. Let $\mathcal{P}$ be a conjunction of non-trivial disequations such that $|\texttt{vars}(P)| = m$ and $X \in \texttt{vars}(P)$. For each disequation $s \not\approx_\alpha t \in \mathcal{P}$, the equation $s \approx_\alpha t$ has at most one solution (modulo $\alpha$-renaming) when the variables distinct from $X$ are considered as constants. Let $\mathcal{S}$ the set of such solutions for all these equations. Since $A$ (the domain of $\mathcal{A}$) is infinite, there exists $a \in \mathcal{A}$ such that $[X/a] \notin \mathcal{S}$. Therefore, $[X/a]$ is a solution for $\mathcal{P}$. Now, consider the problem $\mathcal{P}' = \mathcal{P}[X/a]$ which has $m-1$ variables. The result follows by induction hypothesis.

#### 4.3 Termination

To prove termination we define a measure function for NEPs that strictly decreases with each application of a rule. The measure uses the following auxiliary functions:

**Definition 12 (Auxiliary Functions).** *The function* $\texttt{sizePar}(t)$ *denotes the sum of the sizes of the parameter positions in $t$:*

$$\texttt{sizePar}(t) := \sum_{p_j \in \texttt{PosPar}(t)} |p_j|$$

*where* $\texttt{PosPar}(t) = \{p_j \mid t|_{p_j} = Y_i$ *for some parameter* $Y_i\}$*.*

*Given a disjunction of equations, disequations, freshness, and negated freshness constraints $d = C_1 \vee \ldots \vee C_n$ we define auxiliary functions $\phi_1$ and $\phi_2$ over $d$.*

1. *$\phi_1(d)$ is the number of distinct parameters in $d$.*
2. *$\phi_2(d)$ is the multiset* $\{\texttt{MSP}(C_1), \ldots, \texttt{MSP}(C_n)\}$ *where* $\texttt{MSP}(C)$ *is defined by:*
   *(a)* $\texttt{MSP}(C) = 0$ *if $C$ is an equation or disequation and a member of $C$ is a solved parameter (a parameter $Y$ is* solved *in $d$ if there exists a disequation $Y \not\approx_\alpha u$ in $d$ and $Y$ occurs only once in $d$); or if $C$ is a primitive freshness or a primitive negated freshness constraint;*

*(b) otherwise,* $\mathtt{MSP}(s \approx_\alpha t) = \mathtt{MSP}(s \not\approx_\alpha t) = max(\mathtt{sizePar}(s), \mathtt{sizePar}(t))$ *and* $\mathtt{MSP}(a\#t) = \mathtt{MSP}(a\not\#t) = \mathtt{sizePar}(t)$.

**Definition 13 (Measure).** *Let* $\mathcal{P} = \exists\overline{W}\forall\overline{Y} d_1 \wedge \ldots \wedge d_n$ *be a nominal equational problem in conjunctive normal form.* $\mathcal{P}$ *is measured using the tuple:*

$$\Phi(\mathcal{P}) = (N_u, N_d, \psi_1(\mathcal{P}), M, \psi_2(\mathcal{P})), \text{ where}$$

1. $N_u$ *is the number of free variables that are unsolved in* $\mathcal{P}$*. A variable* $X$ *is* solved *if there is an equation* $X \approx_\alpha t$ *and* $X$ *occurs only once in* $\mathcal{P}$*.*
2. $N_d$ *is a multiset that contains for each disjunction* $d_i$ *in* $\mathcal{P}$ *the number of variables that are not d-solved in* $d_i$*.*
   *A variable* $X$ *is d-solved in* $d_i$ *if* $d_i = X \not\approx_\alpha t \vee Q$ *and* $X$ *does not occur in* $Q$*.*
3. $\Psi_1(\mathcal{P})$ *is the multiset* $\{(\phi_1(d_1), \phi_2(d_1)), \ldots, (\phi_1(d_n), \phi_2(d_n))\}$
4. $M$ *is the multiset* $\{M(d_1), \ldots, M(d_n)\}$ *where* $M(d)$ *is the multiset of sizes of the constraints in d. The size of a constraint is the size of its largest member, or 0 if it has a solved variable or it is a primitive (negated) freshness.*
5. $\Psi_2(\mathcal{P})$ *is the total size of* $\mathcal{P}$ *(that is, the number of function symbols, atoms, variables, quantifiers, conjunctions, disjunctions,* $\top$*,* $\bot$ *in* $\mathcal{P}$*.*

Using this measure we can prove the termination of the simplification process.

**Theorem 3.** *The procedure defined in Section 4 for application of rules, expressed as* $\mathfrak{R} := \mathcal{R}_1\mathcal{R}_2 \ldots \mathcal{R}_9$*, terminates.*

### 5 Nominal Equational Solved Forms

We have shown that the simplification process terminates and each application of the transformation rules preserves solutions. We now characterise the normal forms, called *solved forms*. Intuitively, solved forms are simple enough that one can easily extract solutions from it. A first example of well-known solved form is that of *unification solved form*: a conjunction of equations $X_i = t_i$ such that each $X_i$ occurs only once. It directly represents a solution mapping $X_i \mapsto t_i$.

We show in Theorem 4 existence of solutions for certain solved forms, and in Theorem 5 we prove that our procedure is complete with respect to solved forms.

**Definition 14 (Solved Forms).**

1. *A* NEP $\mathcal{P}$ *is in* parameterless solved form *if it contains no universal quantifiers.*
2. *A* NEP *is a* definition with constraints *if it is* $\top, \bot$ *or a conjunction of the form*

$$\mathcal{P} = \exists\overline{W} : \left(\bigwedge_{i=1}^{n} Z_i \approx_\alpha t_i\right) \wedge \left(\bigwedge_{j=1}^{m} Z'_j \not\approx_\alpha v_j\right) \wedge \left(\bigwedge_{l=1}^{p} C_l\right),$$

*such that:*
   - *each* $Z_i$ *occurs only once in* $\mathcal{P}$*;*
   - *each* $Z'_j$ *is syntactically different from* $v_j$*; and*

– *each $C_l$ is either a positive, $a\#X$, or negative, $a\not\#X$, freshness constraint such that each pair $a, X$ occurs at most once in $\mathcal{P}$.*

3. *A* NEP *is in* unification solved form *if it is a definition with constraints which does not contain negative constraints.*

Theorem 4 below shows that a problem reduced to definition with constraints solved form has at least one solution.

**Theorem 4.** *Let $\mathcal{A}$ be any infinite subalgebra of* CORE. *If $\mathcal{P} \not\equiv \perp$ is in definition with constraints solved form, then it has at least one solution.*

*Proof.* First assume $\mathcal{P}$ is in unification solved form (see Definition 14). Let $\nabla$ be the context containing all constraints $C_l$ occurring in $\mathcal{P}$. Furthermore, define the substitution $\sigma$ that assigns to each free variable $X_i$ the term $t_i$, and the substitution $\delta$ mapping each existential variable $W_k$ to $t_k$. Then $[\![\nabla\sigma\delta]\!]_\varsigma$, which is equivalent to $[\![\nabla]\!]_{\varsigma\sigma\delta}$ by Lemma 1, is valid in $\mathcal{A}$. Consequently,

$$[\![\nabla \vdash X_i\sigma \approx_\alpha t_i\sigma\delta]\!]_\varsigma \text{ and } [\![\nabla \vdash W_k\delta \approx_\alpha t_k\delta]\!]_\varsigma$$

are valid judgements. So, $\sigma$ is an $\mathcal{A}$-solution of $\mathcal{P}$ with existential witnesses given by $\delta$. In the general case, when $\mathcal{P}$ is in *definition with constraints* solved form containing also negative constraints, the construction is similar. We can guarantee a solution for the disunification part of the problem, $\bigwedge_{j=1}^{m} Z'_j \not\approx_\alpha v_j$, by Lemma 5.

**Definition 15.** *A set $\mathfrak{R}$ of rules for solving nominal equational problems is* complete *w.r.t. a kind of solved forms $S$ if for each $\mathcal{P}$ there exists a family of* NEP*s $\mathcal{Q}_i$ in $S$-solved form such that $\mathcal{P} \overset{*}{\Longrightarrow}_{\mathfrak{R}} \mathcal{Q}_i$ and $\mathcal{S}(\mathcal{P}) = \bigcup_i \mathcal{S}(\mathcal{Q}_i)$.*

The next result states that a NEP's normal form with respect to the simplification rules given in the previous section is a definition with constraints. In particular, all parameters are removed from the problem. The proof is by case analysis, considering all possible occurrences of parameters in a problem.

**Theorem 5 (Completeness).** *Let $\mathcal{A}$ be any infinite subalgebra of* CORE. *Then the rules in Figures 2, 3, and 4 are complete for parameterless solved forms and definition with constraints solved forms.*

### 6 Conclusion

In this paper, we introduced *nominal equational problems* (NEPs) as an extension of standard first-order equational problems to nominal terms which, besides equations and disequations, includes freshness and non-freshness constraints. We proposed a sound and preserving rule-based algorithm to solve NEPs in the nominal ground algebra CORE, and showed that this algorithm is complete for two main types of solved forms: parameterless and definition with constraints. As future work, we aim to investigate the purely equational approach to nominal syntax via the formulation of freshness constraints using fixed-point equations with the И-quantifier [21], as well as the solvability of nominal equational problems in more complex algebras.
